# Low pH reduces the virulence of black band disease on *Orbicella faveolata*

**DOI:** 10.1371/journal.pone.0178869

**Published:** 2017-06-01

**Authors:** Erinn M. Muller, Nicole M. Leporacci, Keir J. Macartney, Alessandra G. Shea, Rachel E. Crane, Emily R. Hall, Kim B. Ritchie

**Affiliations:** 1 Mote Marine Laboratory, Sarasota, Florida, United States of America; 2 Department of Natural Resources Science, University of Rhode Island, Kingston, Rhode Island, United States of America; 3 Department of Molecular, Cellular and Biomedical Sciences, University of New Hampshire, Durham, New Hampshire, United States of America; 4 Department of Geography, University of Hawaii, Honolulu, Hawaii, United States of America; 5 Unity College, Unity, Maine, United States of America; 6 University of South Carolina, Beaufort, South Carolina, United States of America; Academia Sinica, TAIWAN

## Abstract

Black band is a deadly coral disease found worldwide, which may become more virulent as oceanic conditions continue to change. To determine the effects of climate change and ocean acidification on black band disease virulence, *Orbicella faveolata* corals with black band were exposed to different temperature and pH conditions. Results showed a significant decrease in disease progression under low pH (7.7) conditions. Low pH also altered the relative abundance of the bacterial community of the black band disease consortium. Here, there was a significant decrease in *Roseofilum*, the cyanobacterium that typically dominates the black band mat. These results indicate that as oceanic pH decreases so may the virulence of a worldwide coral disease.

## Introduction

Black-band disease is one of the most prevalent and virulent diseases affecting contemporary corals world-wide with progression rates reaching over 2 cm day^-1^ [1–3]. Black band disease is also a generalist disease affecting at least 42 Caribbean species of corals, including both scleractinians and gorgonians [4,5]. The disease is characterized as a microbial assemblage that creates a dark band, ranging from black to red in appearance, which moves across healthy coral tissue, causing mortality and leaving behind bare skeleton [6]. A cyanobacterium often dominates the black band mats, and contains the light-harvesting accessory pigment phycoerythrin, giving black band disease its distinct coloration. The mat consists of several microbial functional groups including sulfide oxidizers, sulfate reducers, heterotrophic bacteria, fungi, and Archaea [3,6,7]. Black band disease functions by creating microenvironments that are anoxic, low in pH, and high in sulfide [8]. The coral animal is unable to survive in this environment and becomes the organic fuel of the black band disease microbial consortium [8,9]. The black band disease consortium, however, requires these microenvironment conditions to survive and thrive. Although black band has been studied for decades, Koch’s postulates, used to determine disease causation, have not been fulfilled for black band disease because of its complex microbial community. Richardson and Kuta [10] as well as Frias-Lopez et al. [11] suggest that the entire black-band community functions as a pathogenic consortium, and that there may be no individual, primary pathogen responsible for black-band disease. Many studies, however, continue to recognize that the cyanobacteria play a critical role in the creation and maintenance of black band disease [9,11,12].

Environmental conditions are often correlated with black band disease dynamics. Numerous studies show a positive correlation between seawater temperature and the prevalence of black band disease [13,14] and global climate change may lead to increases in black band incidence. Previous research indicates progression rates of black band disease were positively correlated with water temperatures [14–16] also suggesting an increase in virulence under global warming conditions. Mechanisms underlying the response of black band to increased temperature are unknown. The consortium of microbes that create black band may thrive under warm water temperature or the coral host may become immunocompromised under high temperature conditions [17–19].

In addition to increasing water temperatures, there is a predicted decrease in oceanic pH under future climate change scenarios[20]. The impact of decreasing pH on black band disease dynamics is unknown. The objectives of the present study were to i) quantify the effects of temperature and pH on the virulence of black band disease infecting *Orbicella faveolata*, ii) determine whether different temperature and pH conditions changed the photochemical efficiency of the coral-host symbiosis, and iii) characterize the change in bacterial communities within the coral host as well as the black band bacterial consortium under different pH and temperature conditions.

## Materials and methods

### Experimental design

The present study was conducted in the outdoor wetlab facilities at Mote Tropical Research Laboratory (TRL) in Summerland Key, Florida from July 10^th^ 2013 to July 26^th^ 2013, for a total of 16 days. Prior to the onset of the experiment, thirty two fragments of *Orbicella faveolata*, each approximately 10 x 10 cm in size, were collected from the Florida Keys National Marine Sanctuary coral rescue nursery (Permit: FKNMS-2013-095), where corals are held and maintained for scientific purposes. Corals were transported to TRL immediately after collection, individually placed within five gallon tanks, and allowed to acclimate for three days. To conduct artificial inoculations of black band disease, samples of active black band mats were collected from naturally existing infections on *O*. *faveolata* at Wonderland Reef (24.54794 N 81.45700 W) using individual sterile plastic blunt-tip 60 ml syringes while on SCUBA. The black band disease samples were stored in a cooler maintained at ambient seawater temperature and transported back to the laboratory for immediate inoculations. Coral fragments were wounded by scraping away tissue from a single polyp of each *O*. *faveolata* colony with a sterile razor blade. Each coral was infected by placing ~ 0.03 grams of the black-band mat directly onto the wound site.

### Experimental system

The seawater source at Mote TRL was a well water system that was naturally low in pH (see Hall et al. 2012 for details), and air was bubbled into two separate holding tanks to create the two pH water treatments (7.7 and 8.1). A set of clear vinyl airline tubing and polyvinyl chloride (PVC) piping was used to transport water from the two regulated holding tanks to the spigot-operated manifolds leading to individual tanks within different raceways. Four total raceways were used and each raceway contained eight individual five gallon tanks for a total of 32 tanks. Each 5 gallon tank contained a single fragment of *O*. *faveolata*, a heater to regulate temperature, a power-head to create water movement, a lid to reduce the air-water interface exchange, and a clear vinyl tube providing regulated pH water from the PVC manifold containing seawater from the appropriate holding tank. Tanks received flow through treatment pH seawater at ~60 ml min^-1^. Individual heaters were placed within each tank to maintain experimental temperatures. All of the raceways were filled half-way with seawater to help insulate and maintain the temperature of the individual five gallon tanks. Half of each of the tanks were randomly selected and treated with low pH seawater and half were treated with control pH seawater (see S1 Fig). The high temperature and low pH conditions were used to create an environment predicted under the Representative Concentration Pathways (RCP) 8.5, business as usual scenario [20]. Ultimately, there were four treatment scenarios with eight replicates each: control temperature and control pH (27.73°C ± 1.61 standard deviation (SD) and 8.21 pH ± 0.20 SD), control temperature and low pH (27.76°C ± 1.42 SD and 7.69 pH ± 0.19 SD), high temperature and low pH (30.58°C ± 1.15 SD and 8.18 pH ± 0.13 SD), and high temperature and control pH (30.28°C ± 1.28 SD and 7.67 pH ± 0.20 SD). The control temperature and control pH treatment acted as the ‘non-stressed’ control group, whereas the high temperature low pH treatment was considered the future case scenario of the tropical reef environment in 2100 [20]. The pH level and temperature of the individual tanks were measured twice daily with a Mettler Toledo Sevengo Pro handheld pH meter (NBS scale) to ensure each tank remained within the desired environmental conditions. The pH meter was calibrated daily using certified reference standards to ensure accuracy of the measurements. Salinity was also measured daily, and was similar among treatments (see S1 Table). Although total alkalinity was not directly measured, the water chemistry of the TRL well-water system has been well quantified at ~4200 μequiv/kg, which, notably, is twice the amount typically found within the seawater of the Florida Keys [21]. Complete characterization of water quality parameters by treatment is provided in S1 Table.

### Black band disease virulence

Once infections were established, the progression rate, or change in area of mortality caused by black band (cm^2^/day), was measured daily for 16 days. Photographs of the black band disease infection within each colony were taken with a ruler held adjacent to the coral. The total area of mortality, defined as the area devoid of living coral tissue, was measured from the photographs. Measurements were quantified using ImageJ (https://imagej.nih.gov/ij/index.html). The progression rate was calculated as the change in area (cm^2^) of mortality between each time step (i.e., day).

### Photochemical efficiency

The photochemical efficiency of each infected coral colony was measured using a pulse amplitude modulation (PAM) fluorometer. The photochemical efficiency, measured as F_v_/F_m_, is a measurement ratio that represents the maximum potential quantum efficiency of photosystem II if all capable reaction centers were open. This measurement can be used as a proxy for photochemical stress of the coral symbiosis. In general, when photochemical stress is high there are fewer open reaction centers available, and the F_v_/F_m_ ratio is lowered. High F_v_/F_m_ measurements reflect healthy and fully functioning photosymbionts within the coral holobiont. PAM measurements were taken at night (under dark adapted conditions) approximately every other night over the 16 day experimental period. Three readings were taken from healthy tissue approximately 3 cm away from the progression of the band and averaged for the primary sampling unit.

### Bacterial community composition by 16S rDNA pyrosequencing

At the conclusion of the experiment, a random subset of colonies was sampled for next generation sequencing analysis of the bacterial community within the coral tissue/mucus and also within the black band disease bacterial consortium. Three colonies were randomly selected from each of the four environmental treatment scenarios and approximately 3 x 3 cm of coral tissue was scraped and collected using a 60 ml plastic blunt tip syringe (N = 12 coral tissue/mucus samples). A 10 ml syringe was used to aspirate as much of the black band disease mat as possible from three random colonies within each treatment scenario (N = 12 black band disease samples). Approximately, six ml of coral tissue/mucus and two ml of the black band disease sample was spun down using a centrifuge set at 10,000 RPMs and concentrated into two ml Eppendorf tubes prior to processing. The pellet was retained for DNA extraction and the supernatant (water) was discarded. Source DNA was extracted from each coral tissue and black band disease sample using the MoBio Powersoil DNA isolation kit with an extended bead-beating time of one hour (MoBio Inc., Carlsbad, CA). The bacterial community of each sample was analyzed using 16S rDNA 454 pyrosequencing. A modified amplicon pyrosequencing (bTEFAP) procedure was performed with 16S universal Eubacterial primers, a modified 27F and 519R primer. Polymerase chain reaction was carried out using a single-step 30 cycle HotStarTaq Plus Master Mix Kit (Qiagen, Valencia, CA) under the following conditions: 94°C for 3 minutes, 28 cycles of 94°C for 30 seconds; 53°C for 40 seconds and 72°C for 1 minute with a final elongation step at 72°C for 5 minutes. Amplicon products from different samples were combined equally and purified using Agencourt Ampure beads (Agencourt Bioscience Corporation, MA, USA). Samples were sequenced via Roche 454 FLX titanium instruments and reagents following manufacturer’s guidelines. The sequence data was processed at MRDNA laboratory (www.mrdnalab.com, Shallowater, TX) using a standardized analysis pipeline developed and implemented by MRDNA laboratory. Briefly, sequences were depleted of barcodes and primers and then short sequences < 200 bp were removed. Sequences with ambiguous base calls as well as sequences with homopolymer runs exceeding 6 bp were also removed. Sequences were then de-noised and chimeras were removed. Operational taxonomic units (OTUs) were defined after removal of singleton sequences, clustering at 3% divergence (97% similarity) [22]. Final OTUs were taxonomically classified using BLASTn against a curated database derived from GreenGenes, RDPII and NCBI (www.ncbi.nlm.nih.gov; http://rdp.cme.msu.edu [23]) and compiled into each taxonomic level using the standard cutoffs identified in S2 Table.

The sequencing data from this study were submitted to GenBank within the National Center for Biotechnology Information (http://www.ncbi.nlm.nih.gov) under Accession numbers KY576964 –KY579321 for 16S rRNA gene pyrosequencing.

### Statistical analyses

The progression rate data were aligned rank transformed in order to meet assumptions of normality, homoscedasticity, and sphericity using the ‘artool’ package in R [24,25]. A repeated measures factorial analysis of variance (ANOVA) was applied to the aligned rank transformed progression rate data to determine differences within independent variables (pH or temperature individually) over time, as well as the interaction of the two variables [26]. A repeated measures factorial analysis of variance was also applied to the photochemical efficiency data to determine whether F_v_/F_m_ values differed because of treatment or through time, or because of interaction effects. No transformation was needed for the photochemical efficiency data to meet the assumptions of parametric tests.

A permutational analysis of variance (PERMANOVA) was used to test for differences in the bacterial communities operational taxonomic units (OTUs) of the black band disease consortium as well as the coral tissue/mucus samples using the ‘vegan’ package in R [25,27]. The multivariate data were plotted using non-metric multidimensional scaling (NMDS) biplots for visualization. Major classes of bacteria within each sample type (coral tissue/mucus or black band mat) were tested for differences among treatments using factorial ANOVAs after meeting parametric assumptions. Posthoc Tukey tests were used to determine differences among treatment groups after a significant omnibus test.

## Results

### Black band disease virulence

**Fig 1:**
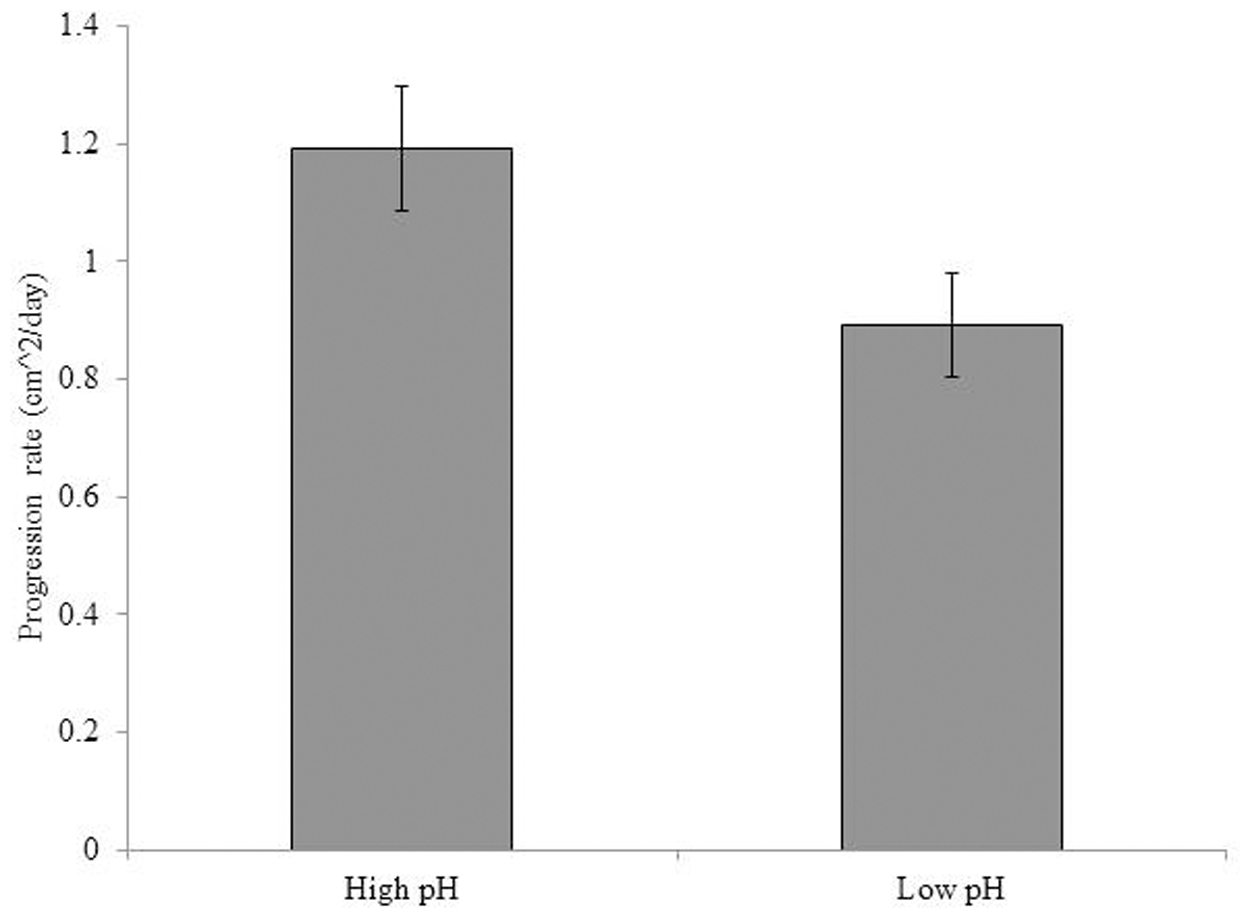
Average progression rates of black band disease on *Orbicella faveolata* under either control (8.1) or low (7.7) pH conditions measured over a 16 day period. Both temperature treatments were pooled within this data since significant differences were not detected. Error bars represent standard error of the mean.

Statistical analyses showed a significant impact of pH on the progression rates of black band disease infecting *O*. *faveolata* with reduced progression rates under the low pH treatment (F_(1,386)_ = 14.175; p<0.001; Fig 1). However, there was no significant impact of temperature on the black band progression rate (F_(1,386)_ = 1.139; p = 0.287, data not shown), nor was a significant interaction detected between pH and temperature (F_(1,386)_ = 0.002; p = 0.966; data not shown).

### Photochemical efficiency

The photochemical efficiency of the coral photosymbiont was not significantly affected by pH (F_(1,178)_ = 0.029, p = 0.864) or temperature treatments (F_(1,178)_ = 1.427; p = 0.234). Photochemical efficiency differed through time as the experiment progressed (F_(1,179)_ = 4.308; p<0.001; S2 Fig). There was no significant interaction between pH and temperature treatments on photochemical efficiency (F_(1,178)_ = 0.023; p = 0.880).

### Bacterial community of coral tissue/mucus

The number of sequence reads within the coral tissue samples ranged from 830 to 8,774 with an average of 3,296 (± 635) reads per sample. There were a total of 2,034 distinct bacterial OTUs identified within the coral tissue samples. A random subset of the lowest number of reads (830) was taken from each sample to account for the variation among samples and to properly compare samples by treatment.

**Fig 2:**
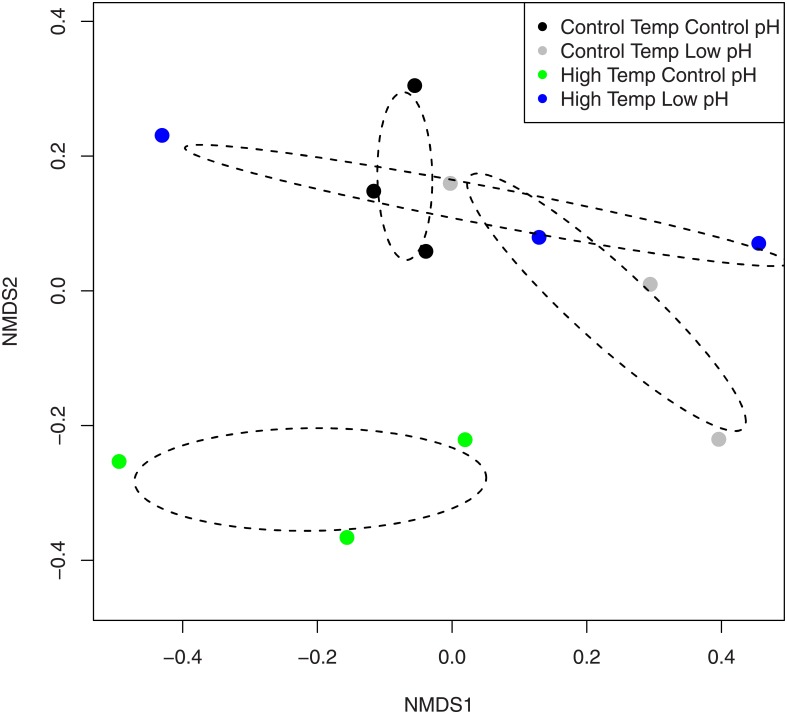
Non-metric multidimensional scaling biplot of the bacterial community OTUs within the coral mucus/tissue of *Orbicella faveolata* infected with black band disease showing the separation of the high temperature (30°C) and control pH (8.1) treatment. Dashed ovals represent the 95% confidence interval of the mean. Stress = 0.13.

**Fig 3:**
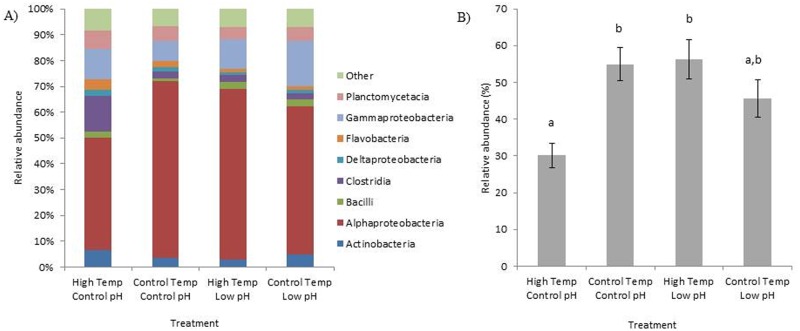
Relative abundance of A) the major classes of bacteria, classes contributing to <3% of the relative abundance were grouped in ‘others’, and B) the bacterial order Rhodobacterales within the tissue/mucus of *Orbicella faveolata* under different temperature and pH treatments. Error bars represent standard error of the mean.

Analysis of the bacterial communities showed that neither pH nor temperature had a direct effect (pH: F_(1,8)_ = 1.166, p = 0.173; temperature: F_(1,8)_ = 1.064, p = 0.309), nor was there a significant interaction between temperature and pH (F_(1,8)_ = 1.362; p = 0.056; Fig 2). The NMDS plot of the bacterial communities within the four treatments, however, indicates that the high temperature and control pH treatment was the most different from all other treatments (Fig 2). Analysis at the bacterial class level showed that the separation of this group was partly because of the Alphaproteobacteria class, which showed a significant interaction between temperature and pH (F_(1,8)_ = 12.674; p = 0.0074). Under high temperature and control pH conditions there was a general reduction in Alphaproteobacteria compared with the other three treatment groups (Fig 3A). Further analyses showed that this decrease was from the significant loss of the order of Rhodobacterales bacteria in the coral tissue when under high temperature and control pH conditions (Fig 3B). The dominant Rhodobacters present within the coral tissue/mucus were most closely identified as *Roseibacterium* (7.99 ±1.70% SE average abundance), *Roseovarius* (8.96±1.53% SE average abundance), and *Ruegeria* species (10.32±1.53% SE average abundance). Flavobacteria and Clostridia bacteria classes were also significantly reduced under low pH conditions (Flavobacteria: F_(1,8)_ = 9.683; p = 0.0144; Clostridia: F_(1,8)_ = 6.231; p = 0.0372).

### Bacterial community of black band disease

The number of sequence reads within the black band disease samples ranged from 1,786 to 19,507 with an average of 10,695 (± 1,666) reads per sample. There were a total of 1,876 distinct bacterial OTUs identified within the black band disease samples. A random subset of the lowest number of reads (1,786) was taken from each sample to account for the sequence read variation and to properly compare samples by treatment.

**Fig 4:**
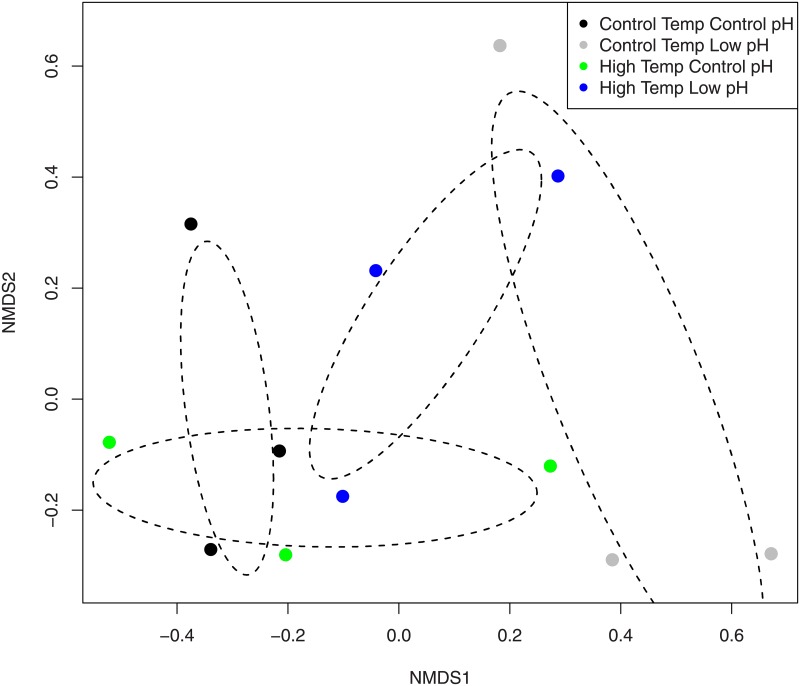
Non-metric multidimensional scaling biplot of the bacterial community OTUs within black band disease on *Orbicella faveolata* that were exposed to four different temperature and pH treatments. Dashed ovals represent the 95% confidence interval of the mean. Stress = 0.10.

**Fig 5:**
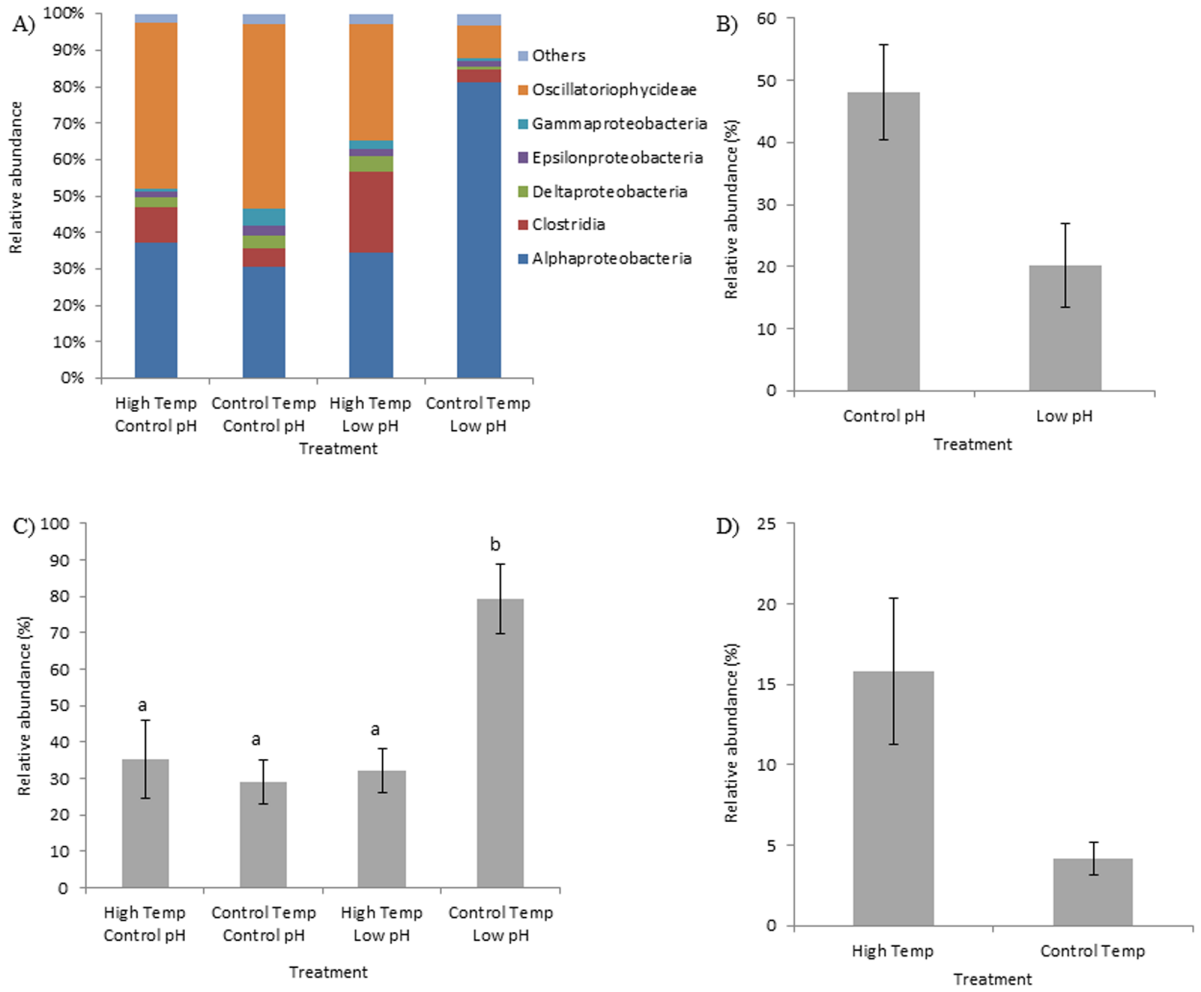
Relative abundances of A) all major classes of bacteria, classes contributing to <3% of the relative abundance were grouped in ‘others’; B) the cyanobacterial genus *Roseofilum*, C) the bacterial order Rhodobacterales, and D) the bacterial class Clostridia within the black band disease consortium infecting *Orbicella faveolata* under different temperature and pH treatments. Error bars represent standard error of the mean. Temperature treatments were pooled within 4B and pH treatments were pooled in 4D because no statistical differences were detected within those treatments for these particular data sets.

There was a significant pH effect on the bacterial community of the black band disease mat (F_(1,8)_ = 2.57, p = 0.039), although there was no temperature effect detected (F_(1,8)_ = 1.76, p = 0.140). A significant interaction between pH and temperature was also detected (F_(1,8)_ = 2.45, p = 0.045). The NMDS plots of the bacterial community, show overlap among all treatments because of high variability within treatments. However, differences in pH treatment seem to fall along the x-axis with a majority of the low pH samples oriented on the right and the control pH samples oriented on the left side of the ordination space (Fig 4). Analysis of the relative abundance of the major bacterial classes showed significant treatment effects on certain classes (Fig 5A). Importantly, there was a significant decrease within the Oscillatoriophycideae class under low pH conditions, regardless of the temperature treatment (F_(1,8)_ = 8.320, p = 0.020; Fig 5B). Alphaproteobacteria showed a significant interaction where abundance increased only under low pH and control temperature conditions (F = 10.965, df = 1, p = 0.012; Fig 5A). Similar to the response detected within the coral mucus, this interaction term was driven by a two-fold increase in the order of Rhodobacterales under these treatment conditions (Fig 5C). There was also a significant interaction term for the class Deltaproteobacteria (F = 13.309, df = 1, p = 0.020; data not shown). Within the black band disease consortium, the abundances of the class Clostridia were significantly higher under high temperature conditions compared with low temperature conditions, regardless of the pH treatment (F = 6.84, df = 1, p = 0.031, Fig 5D).

## Discussion

### Black band disease virulence

Low pH significantly reduced the progression rates of black band disease on *O*. *faveolata*, a response which could have important implications for the prediction of coral-disease dynamics under future ocean acidification conditions. However, the water source used within the present experiment also contained high alkalinity, perhaps confounding the interpretation of the results. Seawater alkalinity is known to buffer the impacts of ocean acidification by providing an excess of proton acceptors within the water column and is often consistent through time. Within the present study, the response of reduced disease progression rates was only observed on corals within the low pH treatment indicating a true, and statistically relevant, treatment effect. Furthermore, previous research showed that most physiological change in corals associated with ocean acidification was primarily from a change in proton (H+) concentrations rather than other carbonate chemistry parameters such as total alkalinity [28]. Therefore, the reduced progression rates of black band disease may occur under low pH conditions regardless of the alkalinity; however, further research is needed to test this hypothesis. Interestingly, another study also suggests a decreased virulence of a crustose coralline algae disease under low pH conditions[29].

Statistical analyses indicated that there was no temperature effect on the black band disease progression rates. An additional short-term study also showed that temperature did not influence the progression rate of black band disease, whereas light was the primary driver [30]. However, the general trend of increasing progression rates with temperature was observed within the Sato et al. [30] seven day study. Similarly, the lack of a temperature response in the present experiment may have resulted from the short term duration of exposure for only 16 days after initial infection. Most other studies observing temperature effects have experimented with naturally occurring and well established infections [14,16]. The progression rates of black band disease on corals within the high temperature treatment were becoming apparent through time. We predict that a response effect to temperature would have become evident with increased exposure time.

### Photochemical efficiency

There was no effect of either temperature or pH on the photochemical efficiency the algal symbionts within the experimental corals. Other studies often show a reduced photochemical efficiency after corals are exposed to prolonged periods of high water temperature, which results in the dysbiosis between the coral host and the photosymbiotic algae that reside within their tissues [31]. Additionally, low pH has caused reef-building corals to bleach under experimental conditions [32]. The present study, however, may not have provided the length of exposure needed to elicit a response. Alternatively, these corals may be generally more resilient to environmental change, perhaps because they were collected from the coral rescue nursery. Here, the corals were held underneath boat docks where light levels were low and turbidity was high. Although no direct water quality measurements were taken, this unique nearshore environment may have influenced the physiology of the corals within the present experiment. For example, previous exposure to high temperature has the ability to increase coral resilience to subsequent high temperature events, at least for some species [33]. The lack of a physiological response, however, indicates that the change in progression rates of the black band disease under low pH conditions was not related to the health state of the host. The photochemical efficiency did change through time, which showed a general increasing trend. These data, again, suggests that the physiological response of the symbiosis between the symbiotic algae and the coral host remained intact and may even have improved because of the experimental conditions.

### Bacterial community of coral tissue/mucus

There was no significant difference in the bacterial community of the coral tissue/mucus among the different treatments suggesting that the change in black band virulence under low pH conditions cannot be explained by the bacterial community of the host. One particular treatment, the high temperature and control pH treatment, however, did separate out distinctly from the other three treatments within the NMDS biplot. An examination of the major bacterial classes show decreased levels of Rhodobacters under the high temperature and control pH treatment with a concomitant increase in Clostridiales and Flavobacteria. Rhodobacters have been associated with tissue-loss diseases within corals [34]. For example, an increase in Rhodobacter bacteria was detected within samples of the coral disease white plague on *Orbicella faveolata*, but was also found within healthy corals indicating a potential increase of opportunistic commensals under certain conditions [35]. High levels of Alphaproteobacteria were also detected within two different coral species showing signs of white plague, *Diploria strigosa* and *Siderastrea siderea*, which was primarily the result of increased abundances of Rhodobacters [36]. Alternatively, some genera of Rhodobacters, such as *Roseobacter*, have been identified as beneficial bacteria, important for the settlement and general health of the coral host [37]. However, within the present study *Roseobacter* was found in low abundances within the corals sampled regardless of the treatment condition. Within the present study, a reduction of Rhodobacters only under high temperature and control pH conditions may suggest sensitivity to temperature, which is then mitigated under low pH conditions.

There were also significantly more Flavobacteria and Clostridiales bacteria within the coral tissue/mucus under control pH compared with low pH conditions. However, the increased abundance of Flavobacteria and Clostridiales under high temperature and control pH conditions is likely driving these statistical results (Fig 3A). Therefore, this response may be simply an increase in growth of these potential pathogenic bacteria under high temperature conditions. Flavobacteria have been implicated in a coral disease outbreak on *Montipora aequituberculata* [38] and associated with white plague-affected *Mussismilia* corals [39]. Clostridiales bacteria also have been associated with several white diseases of corals [34,35]. Similar to Rhodobacters, an increase in these bacteria only under high temperature and control pH conditions, and not within the high temperature and low pH conditions, may indicate that low pH mitigates temperature effects.

### Bacterial community of black band disease

The present study showed that low pH reduced the abundance of the bacterial class Oscillatoriophycidaea, the cyanobacterium that often dominates black band disease. The microenvironment of the black band disease consortium itself creates a low pH, low oxygen, and high sulfide rich area at the interface between the band and the coral host tissue [6]. The cyanobacteria of black band, therefore, are routinely exposed to low pH conditions within the host, but appeared negatively affected by the addition of low pH conditions within the external environment of the present experiment. Cyanobacteria have the ability to regulate internal pH to an extent, although low pH leads to the acidification of the cytoplasm after thresholds are exceeded [40]. Previous research also indicates that low pH reduces cyanobacterial growth rates [41].

Generally, photosynthetic organisms, such as cyanobacteria, are predicted to thrive under ocean acidification conditions, which includes a low pH environment [42]. High levels of carbon dioxide suspended within the water may increase the rate of photosynthesis, thus providing the potential to increase productivity [43]. Although ocean acidification does include reduced pH, the high alkalinity and thus the high *p*CO_2_ levels within our treatments (see S1 Table), limit comparability. Still, even within the context of ocean acidification the results of several studies show variability in the physiological responses of cyanobacteria to low pH. For example, certain cyanobacterial groups such as *Synechoccocus* have shown higher growth rates under low pH conditions [44], as well as reduced growth rates [45]. Similarly, the *Trichodesmium* spp. of cyanobacteria showed reduced nitrogen fixation under low pH conditions, but only when iron was limited [46] Other studies indicate *Trichodesmium* spp. increase both carbon and nitrogen fixation under low pH conditions regardless of iron concentrations [47,48]. To complicate matters further, cyanobacteria that interact in complex assemblages, such as those found in biofilms, may be outcompeted by other photosynthetic organisms under low pH conditions [49]. In microbial biofilms, low pH has reduced the abundance and diversity of cyanobacteria [50–53]. For example, Hassenrück et al. [52] showed that under low pH conditions, the bacterial epiphytic community of seagrasses had a reduced abundance and diversity of cyanobacteria. In the present study, the Oscillatoriophycideae class primarily consisted of the cyanobacteria genus *Roseofilum* (previously named *Oscillatoria*), which dominates the black band disease mat [54]. Arotsker et al. [55] showed that the most transcribed gene in the band consortium was cyanobacterial adenosylhomocysteinase, which is involved in cyanotoxin production and is a large contributor to the virulence of black band disease [56–58]. The cyanobacteria found within black band are indeed interacting with a complex community of other microorganisms [1,8,59]. The many other microbes that create the black band disease mat may be out-competing the cyanobacteria under low pH conditions, resulting in reduced abundances. However, whether the reduced pH is directly influencing the cyanobacteria or another component of the microbial consortium, which ultimately leads to the reduction of cyanobacteria, is unknown. Furthermore, additional studies are needed to determine whether ocean acidification, rather than just reduced pH *per se*, similarly affects the bacterial consortium of black band disease.

A different response of the Alphaproteobacteria bacterial class was observed within the black band disease consortium compared with the bacterial community of the coral tissue/mucus. Here, significant increases in the Alphaproteobacteria were present only under the control temperature low pH treatment scenario. Again, Rhodobacteriales were the dominant group within this heterotrophic bacterial class. The differing responses of the bacterial classes from the coral tissue/mucus compared with the black band disease consortium emphasizes the important role of the environment and the complex interactions among bacterial groups found within different hosts and assemblages (i.e., within an invertebrate host versus a cyanobacterial-dominated mat). Temperature effects were detected within the bacterial class Clostridia within the black band consortium, where high temperatures increased the relative abundance of Clostridia. As mentioned previously, this bacterial class is linked to tissue-loss diseases within scleractinian corals, and flourishes under high temperatures [34,35]. An increase within this bacterial class was also observed within the coral tissue/mucus under the high temperature control pH treatment.

## Conclusions

The progression rate of black band disease was significantly reduced under low pH conditions, which was likely not a result of the physiological state or the bacterial community of the coral host. Analysis of the bacterial community of the black band mat showed a significant reduction in the *Roseofilum* cyanobacteria, a primary pathogenic agent of the black band disease consortium. The present study indicates that pH can significantly influence the community structure of the black band disease consortium, which results in a decreased progression rate of the disease. Therefore, at least under certain conditions, low pH could reduce the virulence of a worldwide coral disease.

## Supporting information

S1 Fig Schematic diagram of the experimental design within the present study. The large blue rectangles represent the 27 C raceway treatments and the large red rectangles represent the 30C raceway treatments. The small black rectangles represent the 5 gallon glass aquaria that each contain a single colony of *Orbicella faveolata* (green circles) infected with black band (black circles). The color within each aquaria represents the different pH treatmtents; green represents 8.1 pH and the peach represents 7.7 pH. The distribution of pH treatments were randomly distributed among tanks. Each aquaria also contained a heater to maintain temperature within the tank and a powerhead to maintain water flow.
(DOCX)

S2 Fig Photochemical efficiency, measured as Yield (F_v_/F_m_) of *Orbicella faveolata* fragments infected with black band disease over the 16 day experiment. Data includes all treatment conditions. Error bars represent standard error of the mean.
(DOCX)

S1 Table Carbonate parameters of treatment water (all data from this experiment with the exception of total alkalinity). *p*CO_2_ and aragonite saturation values were determined using CO_2_SYS. Mean (standard deviation). n = 400 for all parameters except TA (n = 16).
(DOCX)

S2 Table Standardized grouping information to identify each operational taxonomic unit to the most accurate taxonomic level. (DOCX)
