# Supplementary material for: Low pH reduces the virulence of black band disease on Orbicella faveolata
Source: PLoS One. 2017 Jun 1;12(6):e0178869. doi: 10.1371/journal.pone.0178869 (PMC5453599; doi:10.1371/journal.pone.0178869)
Supplement: S1 Fig — The large blue rectangles represent the 27 C raceway treatments and the large red rectangles represent the 30C raceway treatments. The small black rectangles represent the 5 gallon glass aquaria that each contain a single colony of Orbicella faveolata (green circles) infected with black band (black circles). The color within each aquaria represents the different pH treatmtents; green represents 8.1 pH and the peach represents 7.7 pH. The distribution of pH treatments were randomly distributed among tanks. Each aquaria also contained a heater to maintain temperature within the tank and a powerhead to maintain water flow. (DOCX) [file pone.0178869.s001.docx]

**S1 Fig. Schematic diagram of the experimental design within the present study.** The large blue rectangles represent the 27° C raceway treatments and the large red rectangles represent the 30° C raceway treatments. The small black rectangles represent the 5 gallon glass aquaria that each contain a single colony of *Orbicella faveolata* (green circles) infected with black band (black circles). The color within each aquaria represents the different pH treatmtents; green represents 8.1 pH and the peach represents 7.7 pH. The distribution of pH treatments were randomly distributed among tanks. Each aquaria also contained a heater to maintain temperature within the tank and a powerhead to maintain water flow.
