# Supplementary material for: Low pH reduces the virulence of black band disease on Orbicella faveolata
Source: PLoS One. 2017 Jun 1;12(6):e0178869. doi: 10.1371/journal.pone.0178869 (PMC5453599; doi:10.1371/journal.pone.0178869)
Supplement: S2 Fig — Data includes all treatment conditions. Error bars represent standard error of the mean. (DOCX) [file pone.0178869.s002.docx]

**S2 Fig. Photochemical efficiency, measured as Yield (F_v_/F_m_) of *Orbicella faveolata* fragments infected with black band disease over the 16 day experiment.** Data includes all treatment conditions. Error bars represent standard error of the mean.
