# Supplementary material for: Low pH reduces the virulence of black band disease on Orbicella faveolata
Source: PLoS One. 2017 Jun 1;12(6):e0178869. doi: 10.1371/journal.pone.0178869 (PMC5453599; doi:10.1371/journal.pone.0178869)
Supplement: S1 Table — pCO2 and aragonite saturation values were determined using CO2SYS. Mean (standard deviation). n = 400 for all parameters except TA (n = 16). (DOCX) [file pone.0178869.s003.docx]

**S1 Table. Carbonate parameters of treatment water** (all data from this experiment with the exception of total alkalinity). *p*CO_2_ and aragonite saturation values were determined using CO_2_SYS. Mean (standard deviation). n=400 for all parameters except TA (n=16)

| **Treatment** | **Salinity (ppt)** | **Temp (°C)** | **pH_NBS_** | **TA***  **(μmol/kgSW)** | ***p*CO_2_ (μatm)** | **Ω_arag_** |
| --- | --- | --- | --- | --- | --- | --- |
| Control Temp  Control pH | 37.48 (0.26) | 27.73 (1.61) | 8.21 (0.20) | 4202.2  (14.6) | 818.09 (387.96) | 7.39 (1.58) |
| Control Temp  Low pH | 37.52 (0.24) | 27.76  (1.42) | 7.69 (0.19) | 4202.2  14.6) | 3380.65 (1195.25) | 2.79 (1.34) |
| High Temp  Control pH | 37.58  (0.26) | 30.58 (1.15) | 8.18  (0.13) | 4202.2  (14.6) | 1018.94 (454.21) | 7.08 (1.66) |
| Hgh Temp  Low pH | 37.60  (0.24) | 30.28  (1.28) | 7.67  (0.20) | 4202.2  (14.6) | 3871.68 (1542.87) | 2.82 (1.43) |

*Average TA data is from samples taken from system throughout 2013
