# Supplementary material for: Low pH reduces the virulence of black band disease on Orbicella faveolata
Source: PLoS One. 2017 Jun 1;12(6):e0178869. doi: 10.1371/journal.pone.0178869 (PMC5453599; doi:10.1371/journal.pone.0178869)
Supplement: S2 Table — (DOCX) [file pone.0178869.s004.docx]

**S2 Table. Standardized grouping information to identify each operational taxonomic unit to the most accurate taxonomic level.**

| **Identity to reference sequence** | **Identity Designation** |
| --- | --- |
| > 97% | Species |
| Between 97% and 95% | (unclassified Genus) |
| Between 95% and 90% | (unclassified Family) |
| Between 90% and 85% | (unclassified order) |
| Between 85% and 80% | (unclassified class) |
| Between 80% and 77% | (unclassified phylum) |
| < 77% | (unknown) |
